# *IsoNet2* determines cellular structures at submolecular resolution without averaging

**DOI:** 10.21203/rs.3.rs-8363763/v1

**Published:** 2026-01-21

**Authors:** Yun-Tao Liu, Hongcheng Fan, Jonathan Jih, Liam Tran, Xiaoying Zhang, Z. Hong Zhou

**Affiliations:** 1 Department of Microbiology, Immunology, and Molecular Genetics, University of California, Los Angeles (UCLA), Los Angeles, CA 90095, USA.; 2 California NanoSystems Institute, University of California, Los Angeles (UCLA), Los Angeles, CA 90095, USA.; 3 Molecular Biology Institute, University of California, Los Angeles (UCLA), Los Angeles, CA 90095, USA.

## Abstract

We introduce *IsoNet2*, an end-to-end self-supervised deep-learning method that directly reconstructs high-quality 3D densities from cryogenic electron tomograms. A unified network simultaneously performs denoising, contrast transfer function correction, and missing-wedge restoration, achieving ~20 Å resolution without averaging. A feature-rich GUI enables rapid, dataset-specific fine-tuning for end-users. *IsoNet2* resolves domain organization in HIV capsid proteins, tRNA occupancy in individual ribosomes, and *in situ* architectures of mitochondrial respiration-related complexes, enabling atomic-level interpretation of cellular environments.

Two well-established averaging strategies—single-particle analysis (SPA) in cryogenic electron microscopy (cryoEM) and subtomogram averaging (STA) in cryogenic electron tomography (cryoET)—have transformed structural biology by enabling near-atomic-resolution reconstructions of macromolecular complexes. However, both approaches rely on a strong “single-particle” prior: the assumption that molecules being averaged are structurally identical or share a rigid core. This assumption is critical for achieving three objectives central to biological EM structure determination: (i) merging information from differently oriented particles to complete Fourier sampling, (ii) reducing noise through averaging, and (iii) enabling accurate contrast transfer function (CTF) correction. Yet many biologically important systems do not exist in large identical copies in cells. Pleomorphic assemblies, most nucleic acids, lipids, intrinsically disordered regions, phase-separated condensates, and mesoscale molecular organizations therefore remain “averaging-invisible,” largely inaccessible to the current paradigm of high-resolution structure characterization.

Efforts to improve interpretability of raw cryoET tomograms without averaging have included iterative reconstruction algorithms such as SIRT^1^ and SART^2^, which enhance contrast but sacrifice high-frequency detail. Early deep-learning approaches such as *CryoCARE*^3^ introduced *Noise2Noise*^4^ denoising, and the original *IsoNet*^5^ demonstrated that neural networks, when trained on user data, can produce effective missing-wedge compensation. Recent methods, including *DeepDeWedge*^6^ and *CryoLithe*^7^, integrate *Noise2Noise* training with wedge-aware design, but have yet to optimize for recovery of high-resolution 3D information, a necessary precursor to “true” single-particle analysis.

We now present *IsoNet2*, which resolves variation-faithful submolecular features in an authentic sample context, to move molecular structural interpretation beyond the traditional SPA/STA paradigm by eliminating obligate need for particle averaging. To accomplish this, we designed *IsoNet2* to integrate the three core objectives of cryoET reconstruction in a single deep-learning optimization loop (Fig.1a and Extended Data Fig.1), using a “Petronas” architecture (two prongs communicating via bridge, reminiscent of Southeast Asia’s Petronas Towers) that implements (i) accurate missing-wedge compensation to restore Fourier completeness, (ii) *Noise2Noise*-driven denoising, and (iii) network-based CTF correction that sidesteps limitations of classical Wiener deconvolution, thereby facilitating recovery of high-spatial frequencies. Our method operates direct on even–odd tomograms and is implemented via a rich web-technology–based graphical user interface (Fig.1a and Extended Data Fig.2), replete with job submission management, live process monitoring, and user-definable network models and parameters. This enables robust and efficient dataset-specific tuning—an increasingly recognized approach to optimize model performance on small data regimes^8,9^ (as is often the case with unique or rare events visualized in cryoET).

For missing-wedge correction, *IsoNet2* deploys a self-supervised strategy where subtomograms first are randomly extracted, then passed through a neural network in inference mode to produce initial volume predictions. Initial predictions are filtered with corresponding CTF and noise, after which the missing-wedge region of these filtered volumes is integrated with original data to generate missing-wedge–“filled” subtomograms. These “filled” subtomograms contain experimental information in the measured Fourier region and network-predicted information in the missing region. Filled subtomograms are then randomly rotated and used as training targets while their missing-wedge–applied versions serve as inputs, allowing a U-Net–based architecture (Extended Data Figs.1c) to learn missing-wedge restoration across all orientations. Critically, unlike the original *IsoNet* [henceforth *IsoNet1*], which assumed a fixed ±60° tilt range, *IsoNet2* supports per-tomogram wedge geometries, making it suitable for FIB-milled lamellae with variable tilt ranges.

For denoising, *IsoNet2* adopts *Noise2Noise*^2^ techniques using statistically independent half-datasets derived from even–odd movie frames or tilt images. By minimizing discrepancies between the two halves, *IsoNet2* removes uncorrelated noise while preserving signal, similar to implementations in *CryoCARE*^3^, *WARP/M*^10^, and *Topaz-Denoise*^11^. To separate the loss between missing-wedge compensation and *Noise2Noise* denoising, we adapted a masked loss strategy first demonstrated in *DeepDeWedge*^6^.

CTF correction in *IsoNet2* is performed by exploiting the network’s predictive capacity. Namely, the network output is multiplied by experiment-specific CTF before comparison against intrinsically CTF-modulated targets (from “filled” tomograms). This forces the network to output pre-CTF–applied (i.e., CTF-corrected) predictions, avoiding traditional Wiener filtering while recovering information near CTF zeros. To address deviations of low-frequency signal from theoretical CTF behavior, we clamp the CTF curve to unity below the first peak, similar to the “CTF intact first peak” approach in *RELION*^12^. We also implement user-defined B-factor weighting, allowing tailored training to prioritize high- or low-resolution information recovery (Extended Data Fig.1b).

All components—missing-wedge restoration, denoising, and CTF correction—are jointly optimized as differentiable operations in a fully end-to-end training loop. This unified approach delivers substantially improved tomogram quality and reduces over-smoothing effects observed in *DeepDeWedge* reconstructions (Extended Data Fig.3). Unlike *IsoNet1*, *IsoNet2* eliminates iterative training–prediction–regeneration cycles, eliminates explicit particle extraction and external CTF deconvolution, and adopts mixed-precision training^13^, accelerating *IsoNet2* processing by roughly an order of magnitude (Extended Data Fig.1d). The resulting efficiency enables training with substantially larger subtomograms (typically 96^3^ to 128^3^ voxels versus 64^3^ in *IsoNet1*) and an additional down/upsampling layer in the U-Net^14^ architecture, providing a wider receptive field, all while permitting routine use of smaller pixel sizes (~5 Å/pixel versus ~10 Å/pixel in *IsoNet1*) for high resolution reconstructions.

We evaluated *IsoNet2* on an immature HIV virus-like particle dataset (EMPIAR-10164)^15^ previously used to benchmark *IsoNet1* (Fig.1b). These particles feature Gag proteins forming an incomplete spherical lattice of curved, hexagonally packed capsomers. *IsoNet2* dramatically enhanced tomogram clarity, revealing the narrow inter-hexamer gap created by capsid N-terminal domain (CA-NTD) ridges—a feature obscured without CTF correction (Fig.1c). Processed tomograms exhibited near-isotropic resolution, faithfully resolving the Gag lattice across orthogonal views, with lattice defects and local heterogeneity readily apparent (Fig.1d and Supplementary Video 1). In Fourier space, distinct lattice peaks extended to ~22 Å even within the original missing-wedge region (Fig.1e–f), consistent with the ~21 Å median local resolution assessed by *ResMap*^16^ (Extended Data Fig.4). Restored densities further delineated both CA-NTD and CA-CTD domains, the six-helix CA–SP1 bundle beneath each capsomer, and additional density comparable in strength to the capsomer directly beneath the CA–SP1 bundle, likely corresponding to the nucleocapsid domain for which no atomic model exists (Fig.1g–h).

We next applied *IsoNet2* to tomograms of purified 70S ribosomes acquired by *PACEtomo*^17^ (EMPIAR-10985; Extended Data Fig.1a). Processed volumes clearly resolved large and small ribosomal subunits (Fig.2a). Rigid-body fitting a 70S atomic model^17^ accurately placed double-stranded rRNA helices (~20 Å diameter) in density (Fig.2b) in agreement with local resolution estimates (Extended Data Fig.5). Flexible peripheral rRNA extensions—typically lost during subtomogram averaging—were retained (Fig.2b, Extended Data Fig.6, and Supplementary Video 2). Using *ChimeraX* virtual reality tools^18^, we directly inspected individual ribosomes in VR to classify them by native tRNA occupancy at the A, P, and E sites. *IsoNet2* yielded unambiguous tRNA densities, revealing a heterogeneous ribosomal population with A-site–only, P-site–only, A/P, or empty tRNA occupancy states (Fig.2c–h).

Finally, we applied *IsoNet2* to an *in situ* cryoET dataset of *Chlamydomonas reinhardtii* lamellae prepared by cryo-plasma FIB milling (EMPIAR-11830)^19^, analyzing fifteen mitochondria-containing tomograms. Compared to *CryoCARE*, *IsoNet2* reduced missing-wedge artifacts and improved denoising (Extended Data Fig.7). Notably, *IsoNet2*’s ability to produce relatively uniform density quality across entire tomogram volumes permits visualization of all cellular components at a single threshold, creating a “Goodsell-esque”^20^ molecular panorama evocative of David Goodsell’s illustrations that vividly highlights authentic molecular crowding and sociology in cells (Fig.3a, Extended Data Fig.8, and Supplementary Video 3). Macromolecular assemblies, including ribosomes, microtubules, and cristae-studded oxidative phosphorylation machinery, were instantly recognizable in 3D, permitting direct visualization of higher order architecture. For instance, ATP synthase, with stalk and stator regions clearly visible, presents as dimers arranged in semi-helical ribbons along crista ridges, consistent with prior STA^21^ (Fig.3b–c and Extended Data Fig.8b–e). At ridge apices, double rows of adjacent dimers are frequently observed, their spacing likely driving the characteristic 180° membrane curvature. Lastly, *IsoNet2* recovered sufficient detail to resolve three dispersed cytosolic 26S proteasomes^22^—distinguishing the 20S core, 19S regulatory caps, and subunit-level features (Fig.3d and Extended Data Fig.8f)—demonstrating that our network does not rely on copy number in sampled subtomograms to recapitulate accurate 3D structure.

Together, *IsoNet2*’s results demonstrate that a unified deep-learning strategy can achieve high-fidelity structural interpretation direct from raw tomographic data, without the need for particle averaging. Our streamlined GUI encourages robust end-user experimentation to extract optimal model performance tailored for individual data. When combined with predictive tools such as *AlphaFold*^23^ and immersive visualization frameworks in VR/AR, *IsoNet2* moves the field measurably closer to the long-standing goal of interpreting molecular sociology^24^ at near-atomic detail, to ultimately inform construction of comprehensive, full-atom models of cells.

## Methods

### Overview of *IsoNet2*

*IsoNet2* was implemented in Python and runs under the Linux operating system. The software package, including source code and documentation, is available on GitHub (https://github.com/IsoNet-cryoET/IsoNet2). *IsoNet2* uses the PyTorch^26^ deep-learning framework, replacing the TensorFlow^27^ backend previously used in *IsoNet1*.

*IsoNet2* unifies the three essential tasks of cryoEM tomogram reconstruction within a single optimization pipeline: robust missing-wedge compensation restoring Fourier completeness, *Noise2Noise*-based denoising, and a learned contrast transfer function (CTF)-correction module that circumvents Wiener deconvolution. *IsoNet2* also retains all core functionalities from *IsoNet1* for processing tomograms without even–odd splitting—including mask generation, CTF deconvolution, and the full ‘Refine’ workflow. In addition, *IsoNet2* introduces a *CryoCARE*^3^-like *Noise2Noise* denoising module for rapid denoising, which can optionally incorporate the network-based CTF-correction to yield higher-resolution outputs.

The software accepts paired tomograms (even and odd) as input, together with imaging parameters such as electron voltage, spherical aberration, amplitude contrast, defocus values, and tilt range. These parameters are required for CTF correction and missing-wedge correction. The STAR file format used in *IsoNet2* follows the *RELION-5*^28^ standard, ensuring compatibility with established cryoEM processing pipelines.

To generate paired tomograms, when the tilt images are acquired as dose-fractionated movies, even–odd splitting can be performed during motion correction: alternating movie frames (e.g., even vs. odd frames) are separated, motion-corrected, and summed independently, yielding two sets of images with same signal but statistically independent noise. If dose-fractionated movies are not available, even–odd tomograms can instead be generated directly from the tilt series by dividing the tilt images into two interleaved subsets (e.g., tilts 0, 2, 4… and 1, 3, 5…) and reconstructing each subset independently. This produces a pair of tomograms that share the same underlying structure but contain uncorrelated noise, which is essential for *Noise2Noise* learning in *IsoNet2*. We encourage users to perform frame-based even–odd splitting where possible.

### Graphic user interface (GUI)

*IsoNet2* includes a rich desktop graphical user interface (GUI) written in JavaScript, providing an integrated visual workflow for running and managing *IsoNet2* (Extended Data Fig.2). Built with Electron, React, and Node.js, the GUI combines a lightweight desktop front end with a Python backend executed within a Conda environment. It offers tools for dataset organization, parameter configuration, job submission, and real-time process monitoring, with streaming logs and progress visualization. The interface arranges the main processing steps in a left-hand menu, while the central panel shows the program’s live output during a run. Parameter drawers on the right allow users to select input files, adjust basic settings, and submit jobs. Asynchronous inter-process communication (IPC) between the Electron frontend and Python backend ensures responsive interaction even during GPU-intensive computation, supporting user-definable network models and parameter tuning.

### The neural network architecture of *IsoNet2*

**Extended Data Fig. 1 |:**
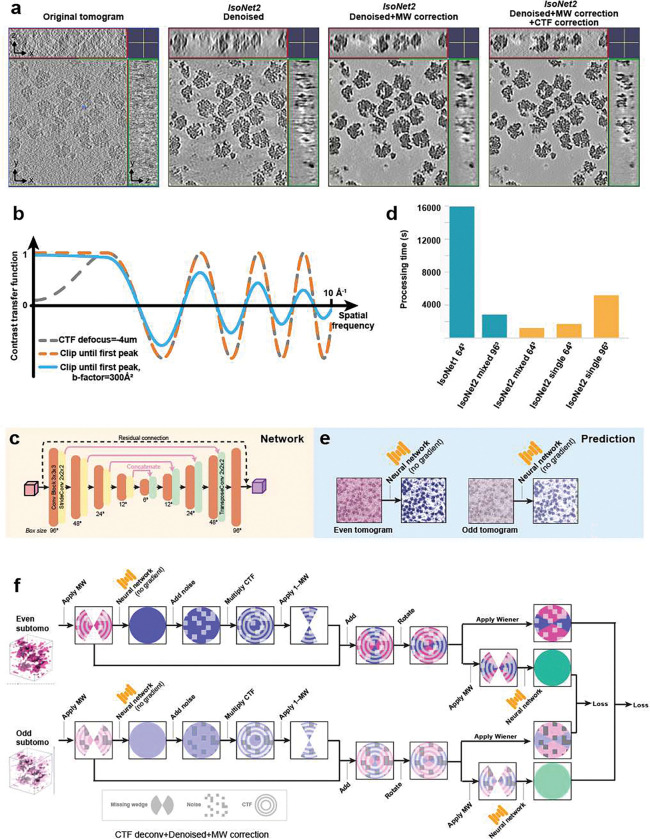
Overview of *IsoNet2* processing. **a,** Sequential improvements in tomogram quality for the ribosome tomograms produced by *IsoNet2*. From left to right: original tomogram, denoised result, denoised + missing-wedge–corrected output, and full denoising + missing-wedge + CTF–corrected reconstruction. **b,** *IsoNet2*’s CTF curve. Low-frequency CTF components are clipped to the first peak, preventing incorrect modulation at very low resolution. Optional B-factor weighted CTF is in blue. **c,** *IsoNet2* network architecture: a 3D U-Net with four down-sampling and up-sampling layers, residual connections, and a larger receptive field (96^3^ input cube). **d,** Processing time comparison between *IsoNet1* and *IsoNet2* under different training modes and cube sizes. Mixed-precision *IsoNet2* achieves ~10× faster computation than *IsoNet1* and enables efficient training on larger boxes. **e,** Prediction pipeline in which even and odd tomograms are independently processed by the trained neural network to generate refined volumes. **f,** *IsoNet2* Refine step using Wiener filter–based CTF correction.

*IsoNet2* employs a 3D U-Net architecture (Extended Data Fig.1c) similar to that of *IsoNet1* and *spIsoNet*^29^, and the basis of which is widely used in biomedical image restoration and segmentation^14^. Each convolutional block contains three 3D convolutional layers (kernel size 3×3×3) with leaky ReLU activations. The encoder path consists of four such blocks, each followed by a strided convolution layer (2×2×2) that halves the spatial dimensions while doubling the number of feature channels. The decoder path mirrors this structure, using transpose convolutions for up-sampling. Skip connections concatenate feature maps of equal resolution between the encoder and decoder paths to preserve high-resolution details. Compared with *IsoNet1*, the default network in *IsoNet2* increases network depth from three to four down-sampling levels while using a lighter 32-filter base.

By default, *IsoNet2* makes use of mixed-precision technology^13^, providing up to a two-fold speed increase without loss of accuracy (Extended Data Fig.1d). The network is represented as $f_{\theta }()$ in the following sessions, where θ denotes all trainable weights updated during training.

### An end-to-end ‘Refine’ implementation

End-to-end refinement is a defining and central process in *IsoNet2*, whereby input tomograms and imaging parameters inform training of a neural network that simultaneously performs denoising, missing-wedge compensation, and network-based CTF correction. Typical training uses ~3,000 subtomograms extracted at a cube size of 96^3^ running from 50–100 epochs.

Unlike *IsoNet1*, which required separate iterative cycles for data updates and network refinement, *IsoNet2* integrates all operations within a single unified optimization loop. This streamlined design eliminates need for intermediate manual steps such as subtomogram extraction and CTF deconvolution, vastly simplifying GUI integration and enabling the entire refinement workflow to be executable by a single command. For example:

isonet.py refine tomograms.star --CTF_mode network

### Refine step 1: Subtomogram preparation

Tomograms are loaded and preprocessed as follows. The mean and standard deviation are computed from the central 64 slices of each tomogram. Subtomograms (default size of 96^3^ voxels and at least 64^3^ voxels) are randomly cropped from even–odd tomograms. The number of subtomograms is user-defined but is recommended to be approximately 3000. Subtomogram extraction is incorporated in the training loop instead of as a separate function like in *IsoNet1*^5^ and *DeepDeWedge*^6^. Prior to network input, cropped subtomograms are normalized by subtracting calculated means and dividing standard deviation values from their corresponding tomogram. When a mask is provided, centers of cropped subtomograms are restricted to masked areas. CTF and optional Wiener filter volumes are also precomputed in this step, which will be described in the CTF section.

### Refine step 2: Generating missing-wedge–filled target subtomograms

Self-supervised learning in *IsoNet2* relies on accurately generating training targets that are faithful to and informed by “ground truth” from experimental data. In order to generate these targets, extracted subtomograms first pass through an initialized neural network (which will be updated in the training loop) to generate initial subtomogram predictions: $f_{\theta }(v_{i}^{0,1})$, where even and odd subtomograms extracted at identical coordinates are denoted as $v_{i}^{0}$ and $v_{i}^{1}$ respectively, and i is the index of the subtomogram.

These initial subtomogram predictions are subsequently added with noise and filtered with CTF that matches the noisy original subtomograms. From these predicted-and-filtered subtomograms, we add information from the missing-wedge region back to the original (missing-wedge–unfilled) subtomograms, generating missing-wedge–filled target subtomograms that are both noisy and CTF modulated. The formula for generating these subtomograms are as follows:

$${\tilde{v}}_{i}^{0,1}\leftarrow FFT^{-1}\left(\left(1-M_{i}\right)\times CTF_{i}\times FFT(f_{\theta }(v_{i}^{0,1})+N_{i})+M_{i}\times FFT(v_{i}^{0,1})\right)$$

where FFT stands for Fast Fourier transform, $M_{i}$ is the missing-wedge mask with zero values in the wedge region and ones elsewhere, and $N_{i}$ is the noise volume that samples from normal distribution N with standard deviation of $\sigma_{i}:\;N_{i}∼N\left(0,\sigma_{i}^{2}\right)$, and $\sigma_{i}^{2}=Var\left(v_{i}^{0}-v_{i}^{1}\right)-Var\left(f_{\theta }\left(v_{i}^{0}\right)-f_{\theta }\left(v_{i}^{1}\right)\right)$. Var means variation.

The neural network model parameters θ used in this step are not updated as no gradients are calculated. This step resembles the ‘predict’ subtomograms step in *IsoNet1*’s refine loop, although *IsoNet1* lacks noise addition and CTF multiplication.

### Refine step 3: Rotating and applying missing-wedge on network input

Missing-wedge information is recovered by learning information from many orientations of target subtomograms. To generate rotated subtomograms and construct paired training samples with and without missing-wedge, we processed missing-wedge–filled target subtomograms ${\tilde{v}}_{i}^{0,1}$ using the formula as follows:

$$u_{i}^{0,1}\leftarrow FFT^{-1}\left(M_{i}\times FFT\left(R_{\varphi_{i}}({\tilde{v}}_{i}^{0,1})\right)\right)$$

where $R_{\varphi_{i}}$ represents rotation of a target subtomogram using a randomly chosen 3D angle $\varphi_{i}\cdot u_{i}^{0,1}$, which contains a missing wedge applied upon the random rotation, is then used as the network input.

### Refine step 4: CTF correction

**Figure 1 |:**
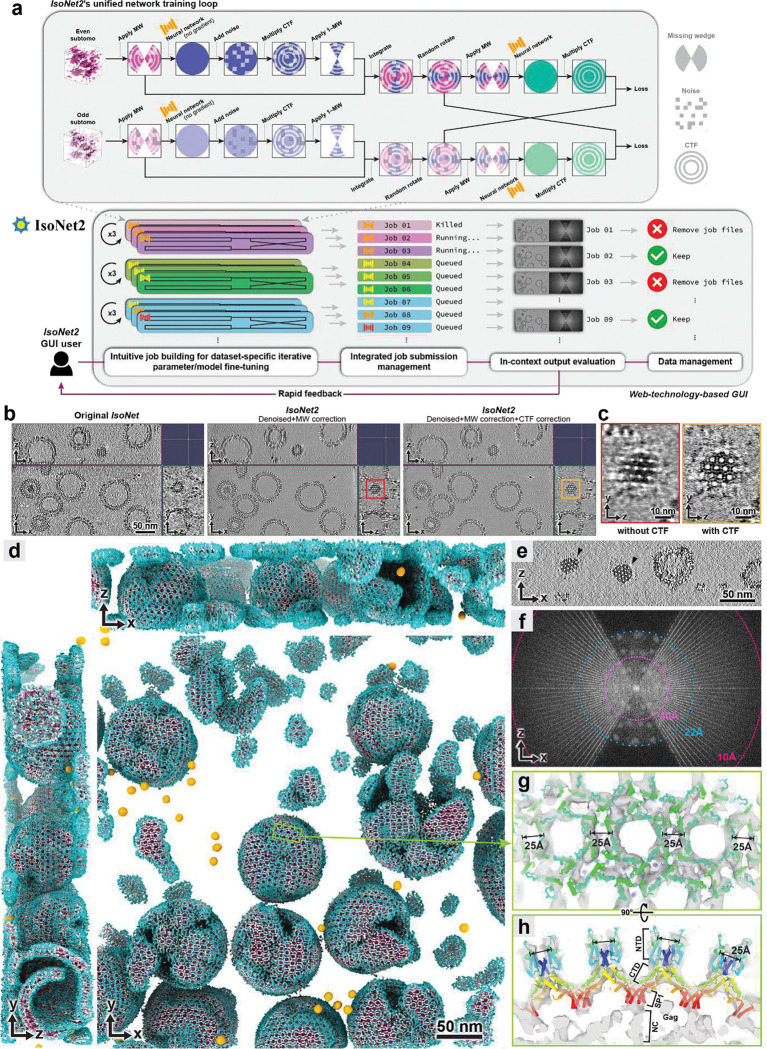
*IsoNet2*’s “Petronas” network architecture and full-featured GUI permit recovery of submolecular features **a**, *IsoNet2*’s bridged dual-prong training scheme operates in a self-supervised, end-to-end loop. A user-friendly GUI facilitates efficient dataset-specific fine-tuning and management of preprocessing and production runs. **b**, Tomographic slices of immature HIV particles (EMPIAR-10164)^15^ processed by *IsoNet1* and *IsoNet2* without/with CTF correction. **c**, Y-Z capsid slices boxed in (**b**) show clear lattice spacing in CTF-corrected. **d**, Orthogonal 3D renderings of *IsoNet2*-processed tomogram (CTF-corrected). Capsid densities colored by distance to lattice surface. Gold spheres are fiducial markers. **e-f**, X-Z slice (**e**) and its Fourier transform (**f**) show peaks (white spots) corresponding to lattice (black arrowheads) recovered in missing-wedge out to ~22 Å. **g-h**, Top (**g**) and cross-section (**h**) views of HIV Gag atomic models (rainbow pipe-and-plank) fit in tomogram density. NTD: N-terminal domain; CTD: C-terminal domain; SP1: spacer peptide 1; NC: nucleocapsid.

*IsoNet2* retains flexibility for users to utilize two approaches for CTF correction: 1. Network-based and 2. Wiener filter–based (*cf.* Fig.1a and Extended Data Fig.1f).

1. *Network-based CTF correction*. After neural network processing of input subtomograms $u_{i}^{0,1}$, missing-wedge–filled outputs are multiplied by CTF. Loss is computed between these CTF-multiplied outputs and their corresponding rotated target subtomograms. This forces the network to output pre-CTF–multiplied (i.e., CTF-corrected) predictions, avoiding traditional Wiener filtering while recovering information near CTF zeros:
$${\mathrm{predicted}}_{i}^{0,1}=FFT^{-1}\left(CTF_{\text{eff}}\times FFT\left(f_{\theta }(u_{i}^{0,1})\right)\right);{\;\mathrm{target}}_{i}^{0,1}=R_{\varphi_{i}}\left({\tilde{v}}_{i}^{1}\right)$$
  At very low-resolution Fourier space regimes (at worse than about 50 Å resolution) of cryoET tomograms, the theoretical CTF does not faithfully reflect the actual CTF modulation present in the data^30^. To address this, we ignore the lowest-resolution region by generating a CTF curve only up to the first CTF peak (Extended Data Fig.1b), similar in concept to “CTF intact first peak” used in *RELION*’s implementation^12^. This modification avoids applying incorrect CTF amplitude corrections at very low resolutions, which helps preserve the initial contrast of original tomograms and leads to reconstructions with better high-resolution details.
  The CTF curve is optionally accompanied by a user-defined B-factor parameter (Extended Data Fig.1b), which controls frequency-dependent signal attenuation applied to the tomogram. Larger B-factor parameters mean signal is attenuated faster per increment of spatial frequency. We recommend setting the B-factor parameter to 200–300 Å^2^ for tomograms of isolated particles and B-factor to 0 Å^2^ for cellular tomograms. Considering “CTF intact first peak” and B-factor, the effective CTF is as follows, where $f_{0}$ is the spatial frequency corresponding to CTF first peak:
$$CTF_{eff}=\left\{\begin{array}{c} CTF(f)\cdot e^{-\left(\frac{B}{4}\right)\cdot f^{2}};f\geq f_{0}\\ e^{-(B/4)\cdot f^{2}};\;f<f_{0} \end{array}\right.$$
2. *Wiener filter–based CTF correction*. In this implementation, and as has been described in *IsoNet1*^5^ and *Warp*^31^, ${Wiener}_{i}=\frac{CTF_{i}}{CTF_{i}^{2}+SSNR^{-1}}$, with an empirical spatial signal-to-noise ratio (SSNR), and is applied to rotated target subtomograms:
$${\mathrm{predicted}}_{i}^{0,1}=f_{\theta }(u_{i}^{0,1});{\;\mathrm{target}}_{i}^{0,1}=FFT^{-1}\left({\mathrm{Wiener}}_{i}\times FFT\left(R_{\varphi_{i}}\left({\tilde{v}}_{i}^{1}\right)\right)\right);$$

### Refine step 5: Compute loss

Network input subtomograms $\left(u_{i}^{0,1}\right)$ from both even and odd tomogram sets are normalized to match their original subtomograms and then passed through the network, and their CTF-multiplied corresponding outputs are used to compute the loss for training. To enable *Noise2Noise*-based denoising, predicted even subtomograms are compared against odd target subtomograms, and vice versa. The final loss is the average of these two comparisons:

$$Loss=\frac{1}{2}\left(\mathrm{Loss}\_\mathrm{func}\left({\mathrm{predicted}}_{i}^{0},{\;\mathrm{target}}_{i}^{1}\right)+\mathrm{Loss}\_\mathrm{func}\left({\mathrm{predicted}}_{i}^{0},{\;\mathrm{target}}_{i}^{1}\right)\right)$$

The loss function can be a standard L1 or L2 loss, or a masked loss as introduced in *DeepDeWedge*. The masked loss is defined as:

$$\begin{array}{l} Loss\_\mathrm{func}\left(x_{i},\right.\left.y_{i}\right)\\ \;={\left\|FFT^{-1}\left(M_{i}R_{\varphi_{i}}\left(M_{i}\right)\times FFT\left(x_{i}-y_{i}\right)\right)\right\|}_{2}^{2}\\ \;+\omega {\left\|FFT^{-1}\left(\left(1-M_{i}\right)R_{\varphi_{i}}\left(M_{i}\right)\times FFT\left(x_{i}-y_{i}\right)\right)\right\|}_{2}^{2} \end{array}$$

where ω is a weighting factor that enables user-modulated weighting of missing-wedge correction versus denoising. Larger ω means prioritizing missing-wedge correction over *Noise2Noise* denoising. In practice, we found that using masked loss with high missing-wedge weight yields better performance with respect to high-resolution feature preservation.

### ‘Predict’ generates final tomogram reconstructions

Upon completion of Refine, the trained network is saved as a “.pt” model file, which can then be applied to conduct inference on the original tomograms or to other tomograms acquired under similar imaging conditions. Even and odd tomograms are predicted independently and combined to produce corrected tomograms (Extended Data Fig.1e). Because processing full-size tomograms easily exceeds GPU memory limits, tomograms are subdivided into smaller 3D chunks. The trained network processes each chunk independently, and outputs are stitched together to generate a final corrected tomogram. In *IsoNet2*, the chunk size is set to match the subtomogram size (default 96^3^ voxels) used during training.

To prevent visible boundaries between chunks—caused by limited context near their edges—we use an overlap-tile strategy^14^, where overlapping regions are jointly predicted and smoothly blended. By default, the overlap width is one quarter of a subtomogram. Because we implemented global normalization on tomograms (versus normalizing individual subtomograms like in *IsoNet1*), patchy artifacts occasionally observed in *IsoNet1* reconstructions are eliminated. Our implementation of Predict can be executed in GUI or with a single command: *isonet.py predict tomograms.star network.pt*

### Other modules in *IsoNet2*

*IsoNet2* also includes an independent *Noise2Noise*-style denoising module similar to *CryoCARE*^3^. This denoising procedure is faster than end-to-end Refine, making it useful for quick dataset assessment and for generating masks. In addition, *IsoNet2*’s standalone denoising can optionally include CTF correction, producing higher-resolution denoised outputs. This denoise module can be performed in GUI or with the command: *isonet.py denoise tomograms.star.*

For tomograms lacking even–odd tomogram pairs, *IsoNet2* preserves deconvolution-based methods and refinement workflow from *IsoNet1*. Therefore, denoising and missing-wedge correction for tomograms without even–odd pairs can still be performed as per *IsoNet1*, albeit with all of *IsoNet2*’s improvements (e.g., workflow handling, elimination of tiling artifacts, etc.).

*IsoNet2* also implements ‘Create Mask’, a mask-generation module that identifies empty regions in tomograms where subtomograms should not be extracted. This improves training efficiency by avoiding unnecessary computation. If mask generation is desired, we recommend mask generation be performed on denoised or CTF deconvolved tomograms using default parameters before the Refine step.

### Processing of HIV dataset EMPIAR-10164

This dataset contains raw cryoET tilt series frames of immature HIV-1 dMACANC VLPs^15^, from which five tomograms were chosen for testing. Raw movie frames were motion-corrected by *MotionCor2*^32^ and split into even and odd frames. Tilt series of even and odd images were then stacked using *IMOD*’s^33^ “newstack” command. Full tilt series alignment and defocus determination were performed with *AreTomo2*^34^ using default parameters. Alignments were then transferred back to *IMOD* for tomogram reconstruction using weighted back projection. Tilt series were 4×-binned prior to tomogram reconstruction, yielding a final pixel size of 5.4 Å/pixel.

For *IsoNet2* processing, we first performed an initial *IsoNet2* Refine with missing-wedge weight (ω) of 200, cube size of 96, and network-based CTF correction with a B-factor of 0 Å^2^, using 2,000 total subtomograms (or 400 subtomograms per tomogram). The resulting network was applied to the original tomograms in a Predict step to generate *IsoNet2*-processed tomograms. These tomograms were then input into our mask generation module using standard deviation percentile 80%, density percentile 50%, and z_crop 0.2.

Output masks were then used for a final Refine step with missing-wedge weight of 200, cube size of 128, and network-based CTF correction with a B-factor of 200 Å^2^, using 5,000 total subtomograms from the five original tomograms for 50 epochs. The final network model was then applied to all five tomograms. To estimate local resolution, a 160-pixel diameter subtomogram containing a spherical virus particle was cropped in 3D using the “crop volume” tool in *ChimeraX*^18^ with contrast inverted by “vop multiply”. Local resolution was estimated for this cropped cubic subtomogram using *ResMap* v1.2^16^.

### Processing of 70S ribosome dataset EMPIAR-10985

This dataset contains cryoET data of 70S ribosomes imaged using *PACEtomo*^25^. Raw movie frames were motion-corrected with *MotionCor2* and split into even and odd frames. Tilt series of even and odd images were stacked by “newstack” in *IMOD*. Full tilt-series alignment and defocus determination was performed in *AreTomo2* under default parameters, and the resulting alignments were imported into *IMOD* for tomogram reconstruction using weighted back projection. All tilt series were 5×-binned, yielding a pixel size of 5.35 Å/pixel.

For *IsoNet2* processing, Refine was performed using the following parameters: missing-wedge weight 200, cube size 96, and network-based CTF correction with a B-factor of 300 Å^2^. A total of 2,400 subtomograms from 80 tomograms were used to train over 50 epochs. Local resolution was estimated using *ResMap* v1.2 on a 160-pixel diameter subtomogram cropped around a centered 70S ribosome particle.

### Processing of FIB-milled cellular cryoET dataset EMPIAR-11830

This dataset contains tilt series acquired from *Chlamydomonas reinhardtii* lamellae prepared by cryo-plasma FIB milling^18^. Defocus files and alignment files were obtained directly from the EMPIAR deposition. Alignments were imported into *IMOD* for tomogram reconstruction using weighted back projection. All tilt series were 4×-binned prior to reconstruction for a final pixel size of 7.84 Å/pixel.

For *IsoNet2* processing, fifteen tomograms containing mitochondria were selected. An initial Refine run was performed using a missing-wedge weight of 20, cube size of 96, and CTF correction with a B-factor of 0 Å^2^. A total of 1,500 subtomograms (100 subtomograms per tomogram) were extracted and used for training. The resulting neural network was then applied to all fifteen tomograms in a Predict job to generate a first round of *IsoNet2*-processed volumes. Mask generation was performed using the default Create Mask settings (standard deviation percentile 50%, density percentile 50%, z-crop 0.2).

Output masks were used in a final *IsoNet2* Refine run with missing-wedge weight 200, cube size 128, and CTF correction with a B-factor of 0 Å^2^. This final refinement used 1,500 subtomograms and trained over 100 epochs. The resulting trained network was applied to the original fifteen tomograms in a final Predict job to yield final *IsoNet2*-processed tomograms. Local resolution was estimated using *ResMap* v1.2 on a cropped subtomogram 128 pixels in diameter.

### Processing of 80S ribosome dataset EMPIAR-10045

This dataset consists of seven tilt series of mammalian 80S ribosomes^35^. Tilt series were split into even and odd sets based on tilt angles. Defocus values were provided alongside the dataset. Tomogram reconstruction was performed in *IMOD* using weighted back projection. All seven tilt series were 6×-binned to obtain a final pixel size of 13.02 Å/pixel.

*IsoNet2* Refine used missing-wedge weight 20, cube size 96, and network-based CTF correction with a B-factor of 0 Å^2^, training on 3,500 subtomograms (500 subtomograms per tomogram) over 50 epochs. The resulting trained network was then applied to original tomograms in a Predict job to obtain final *IsoNet2*-processed tomograms. The *DeepDeWedge*-processed tomogram used for comparison was downloaded from data accompanying the *DeepDeWedge* manuscript.

### Processing cellular cryoET dataset EMPIAR-11078

This dataset contains *in situ* cryoET tilt series of the *Chlamydomonas reinhardtii* ciliary transition zone^36^. Raw movie frames were motion-corrected with *MotionCor2* and split into even and odd frame stacks. Defocus values were obtained from the EMPIAR deposition. Tilt series alignment was performed in *AreTomo2* using default settings, and alignments were imported into *IMOD* for tomogram reconstruction using weighted back projection. All tilt series were 3×-binned to achieve a final pixel size of 10.26 Å/pixel.

*IsoNet2* Refine was performed on eight selected tomograms with missing-wedge weight 20, cube size 96, and network-based CTF correction with a B-factor of 0 Å^2^, training on 2,400 subtomograms (300 subtomograms per tomogram). The resulting model was applied to original tomograms to generate initial *IsoNet2*-processed volumes. Mask generation was performed using *IsoNet2* Create Mask default settings (standard deviation percentile 50%, density percentile 50%, z-crop 0.2).

A second *IsoNet2* Refine was then performed with missing-wedge weight 200, box size 128, and network-based CTF correction with a B-factor of 0 Å^2^, using 2,400 subtomograms over 50 training epochs. The final trained network was used to predict all tomograms. The *DeepDeWedge*-processed tomogram used for comparison was downloaded from data accompanying the *DeepDeWedge* manuscript.

### Tomogram segmentation and rendering

Interpretation of *IsoNet2*-processed 70S ribosome data (EMPIAR-10985) was carried out by fitting the *Escherichia coli* 70S ribosome atomic model^17^ (PDB: 7K00) into ribosomal density using *ChimeraX*’s “fit in map”. Immersive inspection using *ChimeraX*’s AR/VR visualization tools facilitated rapid assessment of model placement in 3D as well as tRNA occupancies in individual ribosomal subunits. After fitting, ribosomes were colored using the “Color Zone” tool to delineate individual particles and highlight subunit-specific features.

**Figure 3 |:**
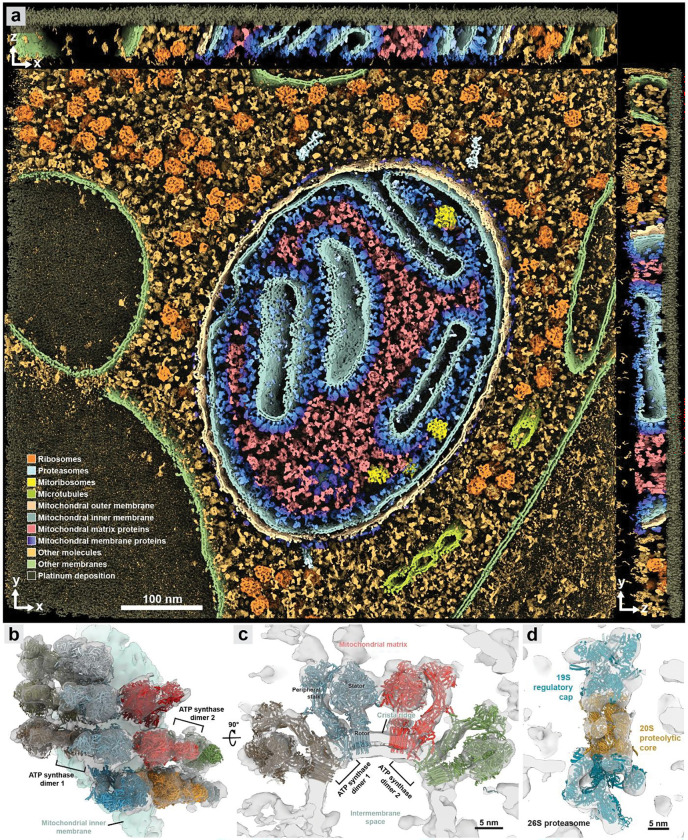
*IsoNet2* enables native visualization of molecular sociology at uniform threshold **a**, A “Goodsell-esque” 3D orthogonal rendering of an *IsoNet2*-processed FIB-milled tomogram of *C. reinhardtii* (EMPIAR-11830)^19^ shows the dense molecular environment in a cellular lamella. **b-c**, Top (**b**) and cross-section (**c**) view of ATP synthase dimers on a crista ridge. Atomic models of ATP synthase (individual dimers labeled “1” and “2”) are fit in unsegmented tomographic density without averaging. **d**, Tomographic density of a cytosolic 26S proteasome fit with atomic model.

Segmentations for the HIV (EMPIAR-10164) and cellular cryoET (EMPIAR-11830) datasets shown in Figures 1 and 3 were performed manually using *Dragonfly* (Comet Group). Viral particles, microtubules, and protein components were segmented using the *Dragonfly*’s Otsu brush, whereas membranes were traced using the line-segment tool. Complete segmentations were exported as TIFF volumes and imported into *ChimeraX* for visualization. Raw densities rather than segmented volumes are displayed in our renderings. To define regions for raw densities, segmentation volumes were low-pass filtered (Gaussian, σ=20 voxels) to generate smooth masks, then binarized in *ChimeraX*. Masks were applied using “vop multiply” to isolate individual components from original tomograms while preserving complementary densities.

For the cellular tomogram in Figure 3, all density was displayed using a single global threshold with distinct colors demarcating structural classes, which results in the dense, Goodsell-style visualization. ATP synthase atomic models^22^ (PDB: 6RD4) were fit directly into our *IsoNet2*-processed tomograms by hand, whereas proteasomes were interpreted using the 26S proteasome model^22^ (PDB: 4CR2).

## Extended Data

**Extended Data Fig. 2 |:**
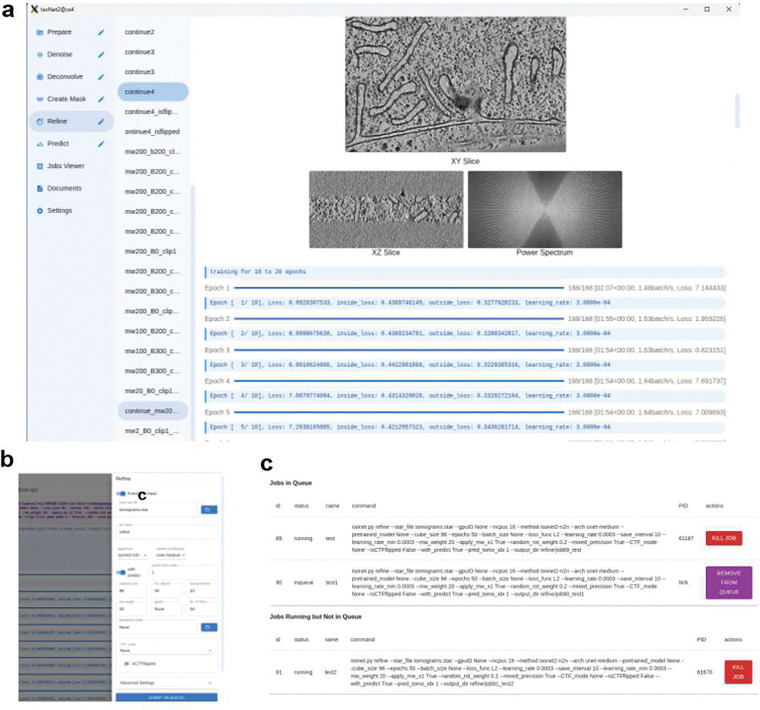
*IsoNet2* graphical user interface and job-management system. **a,** The *IsoNet2* desktop interface, built on modern web-based frameworks, provides an integrated environment for tomogram processing. **b,** The job submission panel. **c,** The *IsoNet2* job manager.

**Extended Data Fig. 3 |:**
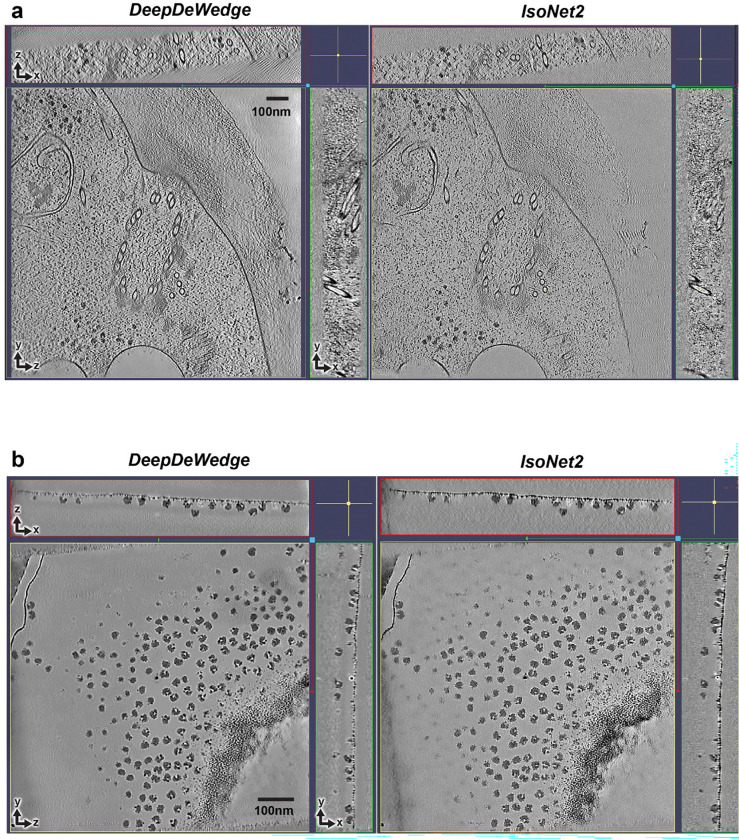
Comparison of *IsoNet2-* and *DeepDeWedge*-processed tomograms **a-b**, Orthogonal slices of a tomogram in EMPIAR-11078 (**a**) showing *C. reinhardtii* ciliary transition zone and a tomogram in EMPIAR-10045 (**b**) showing 80S ribosomes, processed with *DeepDeWedge* and *IsoNet2*.

**Extended Data Fig. 4 |:**
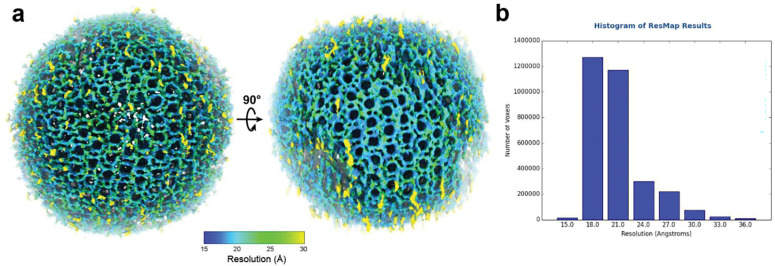
Local resolution estimation of an *IsoNet2*-processed HIV particle **a,** Surface display of a cropped *IsoNet2*-processed HIV particle from EMPIAR-10164 colored by local resolution. **b,** Histogram showing the number of pixels across the map resolution range.

**Extended Data Fig. 5 |:**
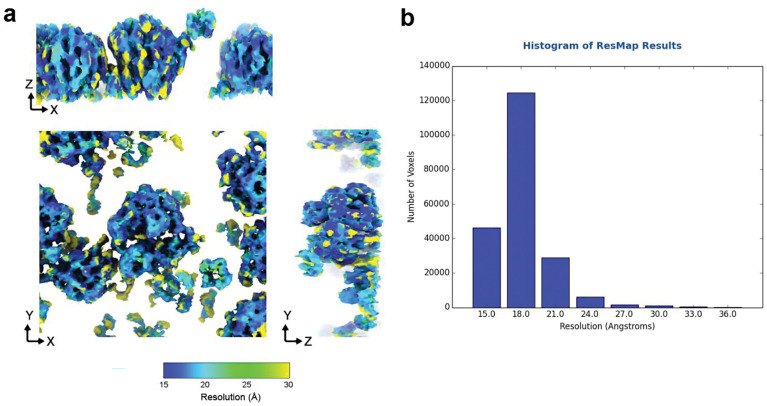
Local resolution estimation for *IsoNet2*-processed 70S ribosomes **a,** Surface rendering of *IsoNet2*-processed 70S ribosomes from EMPIAR-10985 by local resolution. **b,** Histogram showing the number of pixels across the map resolution range.

**Extended Data Fig. 6 |:**
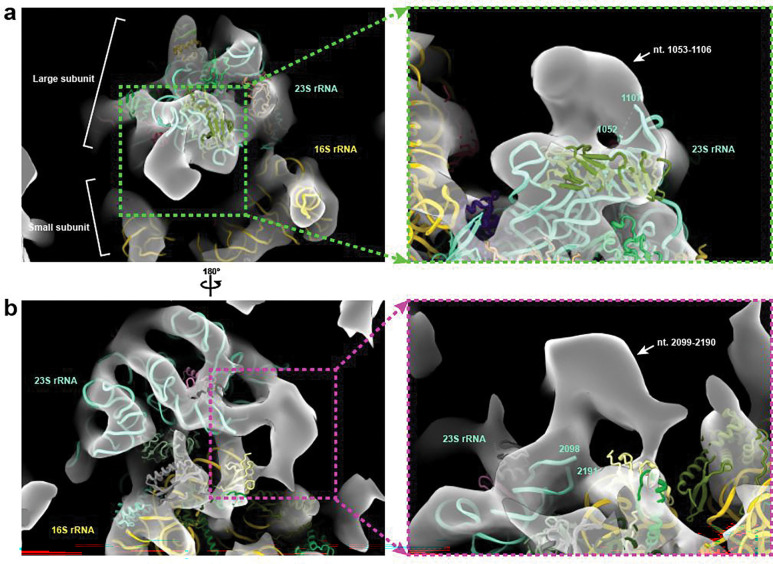
IsoNet2-processed tomograms reveal flexible 70S ribosome features **a-b**, Close up views of 70S ribosome from Fig.2b with *E. coli* 70S ribosome atomic model (PDB: 7K00) docked. Boxed regions highlight flexible regions of large subunit 23S rRNA not visible in averaged 70S structures (hence unmodeled) but visible in *IsoNet2*-processed tomograms, which retain unique, flexible features of individual particles.

**Extended Data Fig. 7 |:**
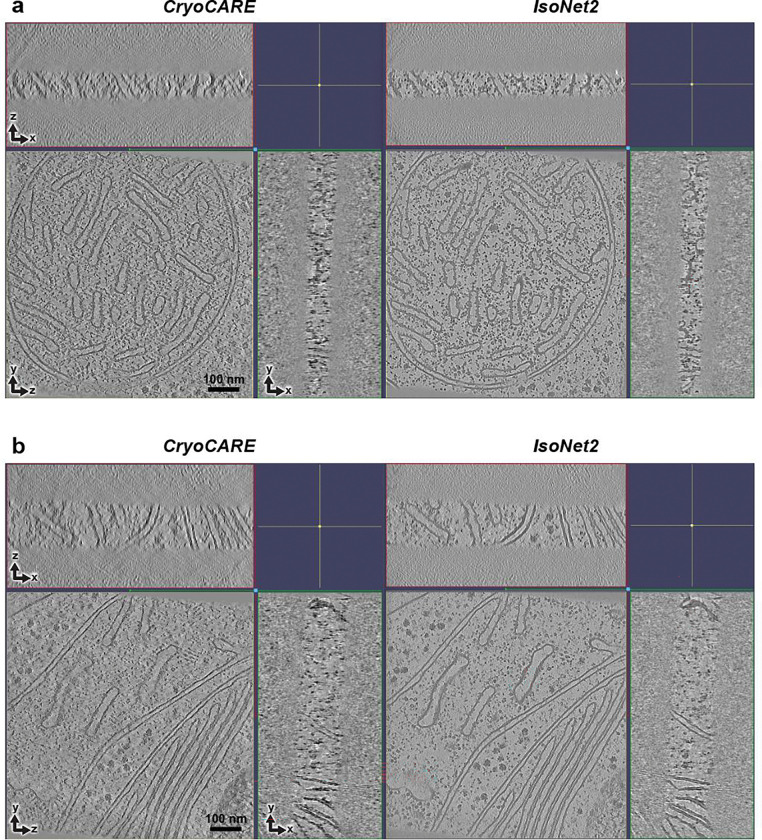
Comparison of *IsoNet2*- and *CryoCARE*-processed tomograms **a-b,** Orthogonal slices of FIB-milled tomograms of *C. reinhardtii* from EMPIAR-11830 processed with *CryoCARE* and *IsoNet2*.

**Extended Data Fig. 8 |:**
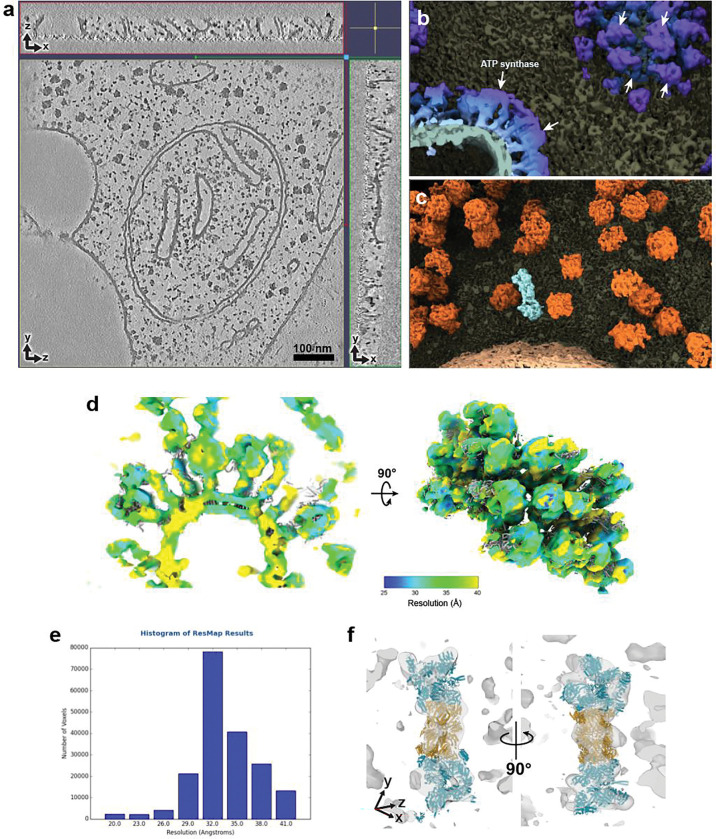
Molecular details and local resolution estimation for FIB-milled cellular tomogram **a-c**, Orthogonal slices (**a**) of the FIB-milled *C. reinhardtii* tomogram shown in Fig. 3 (EMPIAR-11830) with additional 3D views of notable features natively interpretable from the tomogram (**b, c**). **d**, Surface rendering of ATP synthase super-complexes visualized from EMPIAR-11830, colored by local resolution. **e**, Histogram showing the number of pixels across the map resolution range. **f**, Proteasome density (different from Fig. 3D) showing atomic models (PDB: 4CR2) fit into the cryoET density in transparent gray.

## Supplementary Material

Supplementary Files

This is a list of supplementary files associated with this preprint. Click to download.

• SupplementaryVideo1.mp4

• SupplementaryVideo3.mp4

## Figures and Tables

**Figure 2 |:**
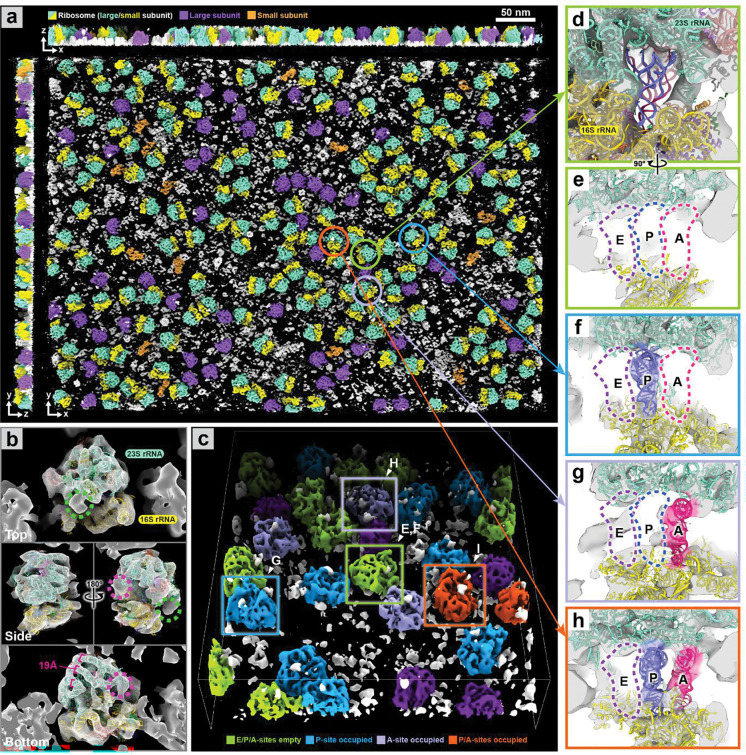
Direct resolution of ribosomal tRNA occupancy by *IsoNet2* **a**, *IsoNet2*-processed tomogram of purified 70S ribosomes (EMPIAR-10985)^25^, colored by ribosomal subunit. **b**, Top, side, and bottom views of unaveraged ribosome docked with PDB-7K00. Dashed circles indicate resolved rRNA density invisible in averaged reconstructions. **c**, Visual classification of ribosomes by tRNA occupancy: empty (green), P-site occupied (blue), A-site occupied (lavender), P/A-site occupied (orange). **d–h**, Observed tRNA binding states in E/P/A sites. In (**d**), E/P/A sites are empty, but tRNA models shown for reference.

## Data Availability

The five cryoET datasets used in this paper are publicly available through EMPIAR: EMPIAR-10164 for HIV particles in Fig.1; EMPIAR-10985 for 70S ribosomes in Fig.2; EMPIAR-11830 for *C. reinhardtii* lamellae prepared by cryo-plasma FIB milling in Fig.3; EMPIAR-11078 for *C. reinhardtii* ciliary transition zone in Extended Data Fig.3; and EMPIAR-10045 for 80S ribosomes in Extended Data Fig.3. All data shown in the figures are available on figshare: 10.6084/m9.figshare.30757889.

## Code availability

The code is available at https://github.com/IsoNet-cryoET/IsoNet2 with both code and tutorial.
